# Charge-Transfer Induced High Efficient Hydrogen Evolution of MoS_2_/graphene Cocatalyst

**DOI:** 10.1038/srep18730

**Published:** 2015-12-21

**Authors:** Honglin Li, Ke Yu, Chao Li, Zheng Tang, Bangjun Guo, Xiang Lei, Hao Fu, Ziqiang Zhu

**Affiliations:** 1 Key Laboratory of Polar Materials and Devices (Ministry of Education of China), Department of Electronic Engineering, East China Normal University, Shanghai 200241, China

## Abstract

The MoS_2_ and reduced graphite oxide (rGO) composite has attracted intensive attention due to its favorable performance as hydrogen evolution reaction (HER) catalyst, but still lacking is the theoretical understanding from a dynamic perspective regarding to the influence of electron transfer, as well as the connection between conductivity and the promoted HER performance. Based on the first-principles calculations, we here clearly reveal how an excess of negative charge density affects the variation of Gibbs free energy (ΔG) and the corresponding HER behavior. It is demonstrated that the electron plays a crucial role in the HER routine. To verify the theoretical analyses, the MoS_2_ and reduced graphite oxide (rGO) composite with well defined 3-dimensional configuration was synthesized via a facile one-step approach for the first time. The experimental data show that the HER performance have a direct link to the conductivity. These findings pave the way for a further developing of 2-dimension based composites for HER applications.

In recent years, the demands for the renewable and clean energy resources gradually become urgent for the growing problems of environmental pollution. Hydrogen, as a clean and efficient fuel source, has been vigorously pursued as a promising candidate for future energy carrier. The traditional way to produce hydrogen involving CO_2_ release and the high temperature reaction condition will be phased out gradually for the related disadvantages^1^, therefore, developing techniques to produce hydrogen from economic and renewable resources can be beneficial to a significant reduction in consumption of fossil fuel and a lower CO_2_ emissions. Recently, massive efforts have been devoted to producing hydrogen by electrochemical or photoelectrocatalytic processes from water splitting^2^,^3^,^4^. So far, many kinds of materials including nickel alloy, carbides, polymeric carbon nitride and transition metal chalcogenides have been attempted to serve as the HER catalysts^5^,^6^,^7^. Among these, the most common catalysts used for HER are noble metals, such as ruthenium, iridium and platinum^8^,^9^. In general electrochemical routines, nickel alloy catalysts present high activity for the HER in alkaline electrolytes, while they are often degraded in acidic solutions. Pt has very small over potential for HER and exhibits excellent electrocatalytic activity, but the scarcity and high prices of these kinds of noble metals prohibit their widespread applications^10^. Therefore, exploring an economical, highly active, chemically stable and abundant materials as HER catalyst to replace the above noble metal and nickel alloy catalysts is of great importance and has attracted a great deal of attention in recent years.

Inspired by the previous discoveries in the hydrogenation and dehydrogenation of petroleum chemicals, a certain amount of metal non-oxides with nanostructures, such as WS_2_, WC, Mo_2_C and MoS_2_^11^,^12^, have been explored for HER applications. Among these catalysts, MoS_2_ nanostructure is a very promising alternative due to its widely distribution, high chemical stability in acid environment and excellent electrocatalytic properties after a certain form of modification^13^. Both theoretical^14^ and experimental^15^ studies found that the HER ability of MoS_2_ mainly derives from the edges of its 2D layers. So far, the main difficulties that impede the extensive use of this material attribute to the restricted surface area, few exposed active edge, and low hierarchy conductivity^16^. The most common ways to overcome the above issues are increasing the active sites of MoS_2_ by resorting to nanoscale or amorphous structures and improving the systematic conductivity through loading the catalyst on highly conductive matrixes. Previously, MoS_2_ cooperated with Au^17^, activated carbon^18^, carbon paper^19^, or graphite^20^ have been fabricated by physical vapor deposition or hydrothermal methods for HER utilization, various overpotentials^21^ and Tafel slopes^15^,^19^ have been attained. With regard to the explanation of the enhancement mechanism for HER performance in composites, the majority of previous works were mainly based on qualitative analyses and the inner mechanisms still need for a further study.

In this work, we studied the HER performance of MoS_2_ cooperated with graphene. Graphene is consisted of sp^2^-hybridized carbon atoms and has attracted many attentions for its excellent conductivity, stability and single-layer configuration. Based on the above features, the combination of MoS_2_ with graphene should be a promising approach to enhance the durability and integral conductivity for the application in HER^22^. We here analyze the adsorption character based on Gibbs free energy form a dynamic perspective under an excess of negative charge density in detail. The obtained results adequately explain the reason why composite structures show a better HER performance and these analytical methods also offer a certain theoretical foundation for a further study of the other HER catalysts. To verify the theoretical analyses, MoS_2_/rGO composite nanostructures were synthesized by a facile hydrothermal method. The experiment data revealed that the HER performance have a link to the conductivity.

## Results

**Figure 1:**
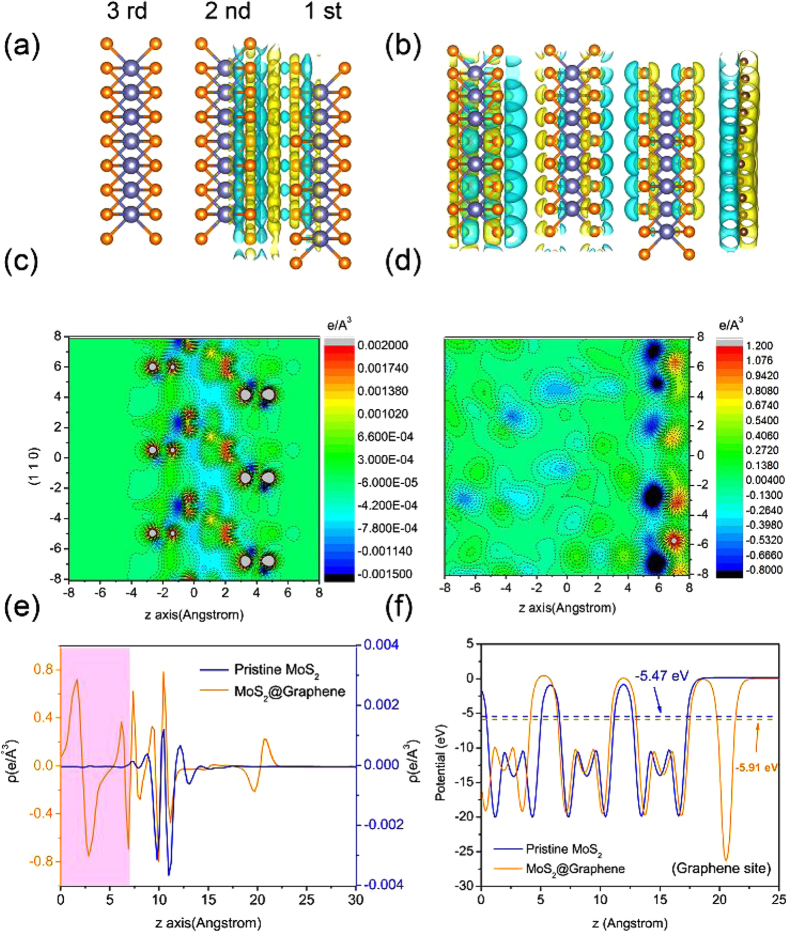
(**a,b**) Charge density difference of pristine MoS_2_ and MoS_2_/graphene composite. Depletion and accumulation spaces are revealed in blue and yellow, respectively. (**c,d**) Plot (110) surfaces of the charge density difference contour for pristine MoS_2_ and MoS_2_/graphene. The unit of charge density is e Å^−3^. (**e**) x–y plane averaged charge difference, with charge density (ρ) on the y-axis and distance (Å) along z-direction on the x-axis. (**f**) Average electrostatic potential of the pristine MoS_2_ and MoS_2_/graphene, in which the vacuum levels are set to zero.

Firstly, charge density difference maps are plotted in Fig. 1a,b to qualitatively elucidate the charge transfer statuses of pristine MoS_2_ and MoS_2_/graphene composite. The charge density difference map represents the charge re-distribution of different portions. All atoms were kept at the same positions as they were in the integral structure. The yellow regions represent charge accumulation while the blue denote charge depletion. Figure 1a indicates that the major charge transfer happens between the 1 st MoS_2_ layer and it proximate 2 nd layer, while the 3 rd MoS_2_ layer is relatively unscathed. This is easy to understand since the absence of strong bonding interactions between MoS_2_ layers and the weak van der Waals (vdW) interactions are expected to play a leading role. To model the MoS_2_/rGO hybrid, we consider the composite system of graphene on bulk structured MoS_2_ surface as presented in Fig. 1b within first-principles density functional theory (DFT). The pristine lattice parameters and geometries are optimized until the forces on each atom are less than 0.01 eV/Å. Then, the unit cells are expanded and combined to form the composite supercell. The geometry of the composite is then allowed to optimize until the forces on each atom are less than 0.02 V/Å. After structural relaxations, graphene and MoS_2_ keep their original planes and hexagonal atomic networks. For the MoS_2_/graphene composite, an obvious difference is that the existence of graphene layer noticeably affect the charge density distribution of the three MoS_2_ layers, in which the charge transfer also appears around the 3 rd layer. Besides, a more remarkable charge transfer between the graphene and the adjacent MoS_2_ layer are found, indicating the presence of a weak ionic interaction more than just the van der Waals that between the stacking layers in the pristine structure. Figure 1c,d show (110) surface of the charge density difference along z-direction, where positive regions represent a gain of charge while negative denote a loss of charge. Clearly, the charge transfer of the pristine structure mainly exists in the first two MoS_2_ layers and there is scarcely any accumulation or depletion around the 3 rd layer. In comparison with the MoS_2_/graphene composite, it shows a much wider interaction scope for the incorporation of graphene layer and the obvious accumulation/depletion regions are also demonstrated around the 3 rd MoS_2_ layer. Figure 1e shows the (x-y) plane averaged charge redistributions in the pristine MoS_2_ and composite structures which are plotted as a function of distance along z-direction. The charge density difference of the pristine structure almost equal to zero around the 3 rd MoS_2_ layer as marked in pink, however, the difference of composite structure fluctuates all along from nearly zero up to the graphene layer, indicating a sustained interaction between the MoS_2_ layers on account of the combination with graphene layer. As illustrated above that the significant charge density difference between the two structures, distinct difference is also reflected in the change of Bader charges. It is calculated that almost ignorable charge transfer of 0.004 e form the 2 nd and 3 rd layers to the 1st MoS_2_ in pristine structure, while this value is 0.03 e from MoS_2_ to graphene, suggesting a stronger interaction between MoS_2_ and graphene. One aspect to note here is that the transfer direction of the electrons in MoS_2_/graphene composite. Experimentally, the work function of the graphene is determined to be 4.57 ± 0.05 eV^23^, while this value is 4.5 V for the bulk structured MoS_2_^24^. Since the work function position of MoS_2_ is higher than that of graphene, the electrons will flow from MoS_2_ to graphene if they contact. Consequently, MoS_2_ will be positively charged and graphene will be negatively charged for the involvement of electrostatic. When the two phases acquire an equalized Fermi level, a built-in electric field directed from MoS_2_ to graphene will be established at the same time. Finally, this electrical coupling to the graphene will facilitate the following rapid electrons transfer from electrode to rGO and then to MoS_2_, forming an excessive negative charge density to enhance the HER performance in the corresponding electrochemical processes.

Previously, Yu *et al.*^25^ studied the HER catalytic activity of MoS_2_ with controlled growth of atomically thin films. Different from the conventional wisdom, they considered that the key to develop the high-efficient catalysts was to increase the hopping efficiency of electrons in the vertical direction based on layer dependent experiments. This is because the potential barriers exist in the interlayer gap of MoS_2_ and the electrons transfer in the z direction is through hopping mechanism^26^. Figure 1f shows the average electrostatic potential of the pristine MoS_2_ and MoS_2_/graphene composite, in which two oscillation patterns of the average potential correspond to the periodicity of the supercell. The averaged potential for the pristine MoS_2_ and MoS_2_/graphene composite are −5.47 and −5.91 eV, respectively. It clearly shows that the averaged potential of composite structure is lower than that of pristine MoS_2_, implying an easier transfer of electrons form electrode to MoS_2_ in the direction perpendicular to the MoS_2_ basal plane for the MoS_2_/graphene composite and an increase of hopping efficiency for electrons in the vertical direction. Therefore, based on the above analyses, it is safe to conclude that an excess of negative charge density can form for the MoS_2_/graphene composite due to the achievement of an increased hopping efficiency for the composite structure when in the corresponding electrochemical processes.

**Figure 2:**
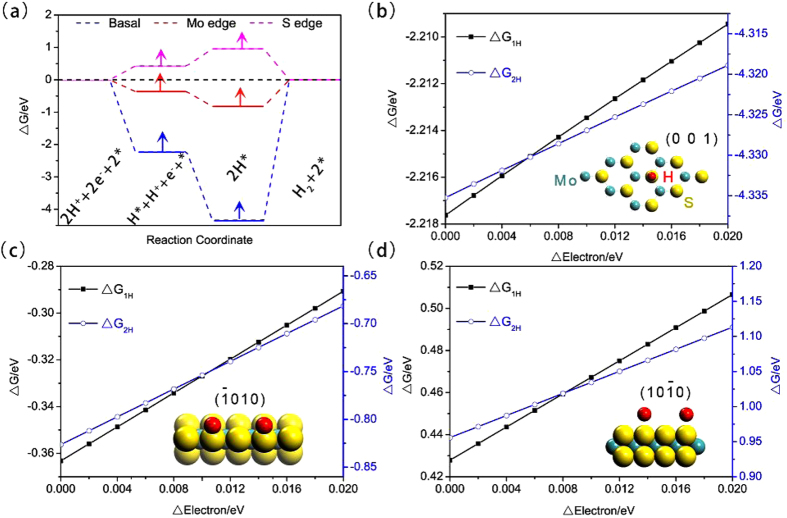
(**a**) ΔG profiles of the HER in the neutral conditions for basal plane, Mo and S edges of MoS_2_. The arrows denote the change direction of Gibbs free energy when extra electrons are added. The adsorption of 1 and 2 hydrogen atoms for the three structures are calculated. (**b–d**) The variation of ΔG profiles versus the amount of extra electrons for the basal plane, Mo and S edges of MoS_2_, respectively. Insert figures show the corresponding adsorbed configurations of H atoms.

The main feature of MoS_2_/graphene composite as analyzed above is that electrons can transfer from graphene to MoS_2_ directionally and the increased hopping efficiency of the system can form an excessive negative charge density. This motivates us to further study what role the excessive negative charge plays other than the enhanced conductivity. The considered atomic structures of basal plane, Mo and S edges are shown in the insert figures of (b–d). One thing to note is that the Mo edge is terminated with single S atom and that the Mo edge is not just a simple truncation of the bulk MoS_2_ structure^27^. The Mo-edge termination with a single S atom matches that of the 50% sulphur covered Mo edge previously observed in model catalysts and predicted by DFT^28^. It is commonly accepted that the criterion for a good HER catalyst is that the Gibbs free energy of adsorbed H is close to the thermo-neutral (i.e., ΔG ≈ 0). Thermodynamically, if the hydrogen adsorption is endothermic, the generation of surface H* would be hindered; while it is too exothermic, the removal of H* to form H_2_ would be difficult^29^. As shown in Fig. 2a, the ΔG of M-edge is more closer to zero and this means that it is the most active toward HER routine in comparison with the other two sites. The ΔG of basal site is much less than zero, implying this kind of site is inert and may not participate in the HER routine. When the extra electrons are added to the three structures, it is obvious that the ΔG of the three structures have a consistent variation trend and are all increased with the increase of extra electrons as shown in Fig. 2b–d. It is remarkable that the ΔG of Mo-edge and basal sites are approaching to thermo-neutral states while S-edges deviating from thermo-neutral. Considering that the optimal value of ΔG should be around 0 eV, which is the ideal state that the binding of H atom is neither too weak nor too strong, it is safe to conclude that the HER activity should be improved when much more electrons involved in the corresponding electrochemical process for the main active site of MoS_2_ for HER is Mo-edge site that approaching to 0 eV. To verify the above analyses, we designed experiments based on MoS_2_/rGO system and intended to change the conductivity of the different structures to simulate various electron-rich levels through the control of hydrothermal reaction temperature.

**Figure 3:**
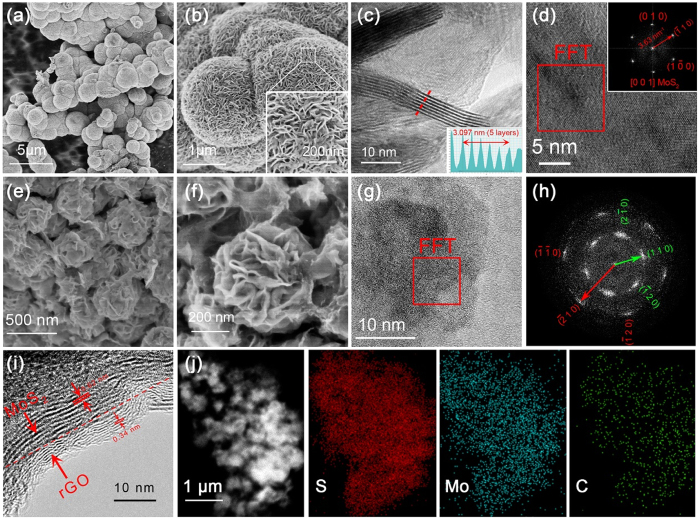
(**a–d**) SEM and HRTEM images of the pristine MoS_2_ under various magnifications. (**e–g**) SEM and TEM images for MG3 composite. (**h**) FFT pattern of the red box shown in (**g**), two sets of patterns marked in green and red can be clearly observed. (**i**) HRTEM image of MG3 composite. (**j**) The EDX mapping images of S, Mo and C.

Figure 3 shows the morphology and microscopic structures of the pristine and MG3 composite characterized by SEM and TEM images. It should be noted that in this section, we only comment MG3 composite since it has the optimal HER performance as discussed below, and thus a detailed analysis based on this sample can enough give the corresponding geometrical configuration of MoS_2_/rGO and elementary composition information. We are aim to synthesize the nanoflowers structured MG composite. The morphology of the pristine MoS_2_ can be clearly observed from Fig. 3a,b, a sequence of SEM images at various magnifications. As presented in Fig. 3a, each MoS_2_ sphere shows an average diameter of 1 to 2 m. The surface of the pristine MoS_2_ has a flowerlike morphology that mainly consisted by 2D nanopetals and the synthesized nanopetals grow in high density, which are freely and tightly aggregated together as exhibited in Fig. 3b. From the insert of Fig. 3b, the high-magnification of an individual MoS_2_ spherical structure, it can be seen that the petals (2D nanosheet) disorderly intersected together. Insert Fig. 3c is the intensity signal along the red line of Fig. 3, it shows that the synthesized pristine MoS_2_ spherical structure grows in a high density way and the pristine MoS_2_ petal tends to stack with a 0.619 nm (3.097 m of 5 layers) interlayer distance.

Figure 3e,f show the SEM images of the synthesized MG3 nanoflowers composed by freely distributed petals. It presents a clear change in comparison with the pristine MoS_2_. The insert Fig. 3b,f of the two structures have the same scale, while the flower-like structures in Fig. 3f are significant more than that of pristine MoS_2_ and grown in a more free and loose way. As analyzed above, the ΔG of M-edge is more closer to zero than that of S-edge and basal site. The sharply increased petals can thus provide much more activity edges and enhance the HER performance. The MoS_2_ in these hybrid agglomerates are mainly consisted by limited-layer MoS_2_ structures and tightly coupled with underlying rGO sheets. In contrast, the TEM image of the MG3 nanostructure shown in Fig. 3g presents scattered and mild appearance of MoS_2_ grown on rGO sheets characterized by varied brightness. These results show that the incorporation of rGO can dramatically suppress the stacking of MoS_2_ to a great extent during its growth progress. Next, the crystallinities of the pristine MoS_2_ and MG3 composite are further characterized by FFT patterns. The FFT pattern of pristine MoS_2_ shown in the insert of Fig. 3d with bright spots denotes highly crystalline property and indexes definitely to MoS_2_. As for the MG3 composite shown in Fig. 3h, two sets of FFT patterns clearly demonstrate the coexistence of rGO and MoS_2_ along (002) direction. One thing to note here is that the FFT pattern of MoS_2_ in MG3 composite turns into arcs, which is clearly different from the well-defined crystal structure of pristine MoS_2_ and this indicates the formation of a quasi-crystalline structure of MG3 composite. We consider the nucleation mechanism on graphene was affected by oxygenated functional groups of GO. In the process of the GO reduced to rGO, MoS_2_ might grow on these sites. Graphene itself is 2D structured, while MoS_2_ has the nature of growing into flower. Both sides result in the confined growth of the corresponding MoS_2_ structure and form this special morphology and the petals are almost parallel to the graphene. Figure 3i further provides the direct evidence of the coexistence of hexagonal MoS_2_ and rGO. The upper left fringes of d = 0.62 nm is in agreement with the (002) plane of MoS_2_, indicating the synthesized petals grow in a crystallization way. The lower right exhibits lattice fringes of 0.34 nm which can be well indexed to (002) plane of 2H graphene (PDF#41-1487). It is apparent that the heterostructure formed between MoS_2_ and rGO in the MG3 composite. From the elemental mapping of Fig. 3j for MG3, it can be seen that the extensive distribution of S, Mo and C. In brief, the modified MoS_2_ nanoflowers are clearly showed from Fig. 3e–i and the SEM/TEM analyses of the MG3 composite prove the formation of the corresponding 3D hierarchical architecture.

**Figure 4:**
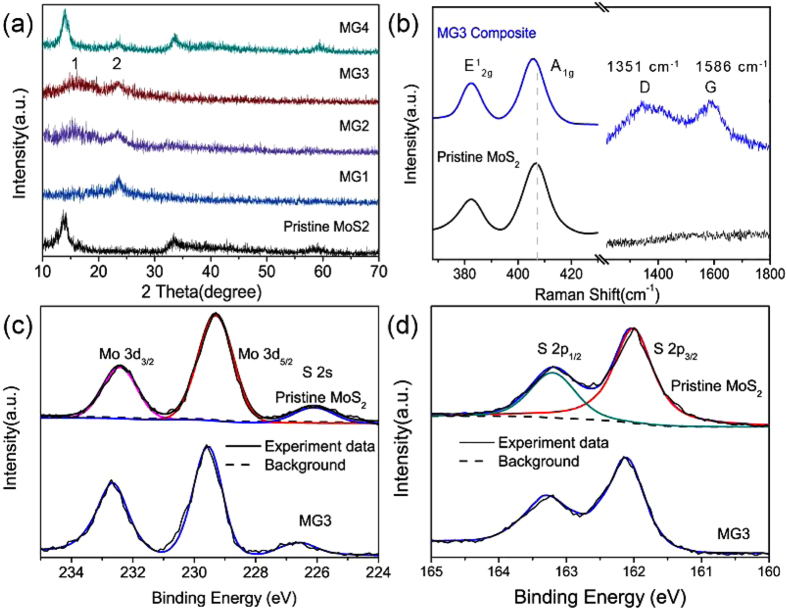
(**a**) XRD patterns of the pristine MoS_2_ and MG1-4 composites. (**b**) Raman spectra of the different vibrational modes for pristine MoS_2_ and MG3 composite. (**c–d**) are XPS spectra of Mo 3d and S 2p.

Figure 4a shows that three peaks of pristine MoS_2_ XRD spectrum are observed, indicating the crystallized MoS_2_ can be obtained through the hydrothermal method at 180 °C with the absence of GO. The three diffraction peaks around 2θ = 14°, 34° and 57° index to (002), (100), and (110) plane of MoS_2_ (JCPDS#37-1492), respectively. The four composites show a progressive crystallinity degree. For MG1-3, the broad peak 1 corresponds to the diffraction of MoS_2_^30^, while the peak 2 around 24° attributes to rGO sheets^31^. Compared with MG3, the pristine MoS_2_ synthesized through the same conditions shows a better crystallinity and this is also accord with the above FFT observation. The significantly decreased diffraction peaks of MoS_2_ for MG3 imply that the incorporation of rGO can restrain the aggregation of natural stacked MoS_2_ during hydrothermal process to a certain degree, leading to a poor crystallinity with a few-layered MoS_2_ nanosheet. Previous reports have proven that the amorphous MoS_2_ presented higher HER activity than that of crystalline MoS_2_ within a given range since the amorphous structure can provide a mass of exposed edges^32^. It is speculated that the MoS_2_ with restrained crystallinity synthesized here can also provide ample edges as active sites due to their confined environment based on rGO network. A clear trend for MG composites is that the intensities of all diffraction peaks of MoS_2_ significantly increase with the increment of temperature and this also accompanies with the appearance of a new peak around 24° ascribed to rGO sheets. Based on the above analyses, it is credible to conclude that the synthesized MG composites at different temperatures have a progressive crystallinity.

Figure 4b displays two peaks at 382 and 407 cm^−1^ corresponding to the E^1^_2g_ and A_1g_ vibrational modes for pristine MoS_2_, respectively. It has been reported that the frequency of A_1g_ mode would present a red-shift as the layer number of MoS_2_ decreases^33^. As shown in Fig. 4b, a slightly red-shift of A_1g_ mode is observed for MG3 composite in comparison with pristine MoS_2_, implying a decreased layer number after combination with rGO, which is also consistent with the above TEM and XRD analyses. The intensities of these two vibrational modes reflect the terminated structure of MoS_2_ and a lower intensity of E^1^_2g_ compared to A_1g_ is associated with an edge-terminated characteristic^34^. At these sites, many unsaturated sulfur atoms serve as active sites and contribute to the improved HER activity. Besides, the Raman spectrum of MG3 shows two other prominent bands at 1351 and 1586 cm^−1^. The two bands correspond very well to the D and G-band of graphene. As previously reported, intensity ratio I_D_/I_G_ is the quality indication of graphene^35^. The nearly 1 value of I_D_/I_G_ agrees well with that of rGO, implying GO was indeed reduced to rGO with some defects or/and disordered structures^36^.

The chemical states of Mo and S in the pristine and MG3 are further investigated by XPS. The XPS spectrum of Mo 3d in pristine MoS_2_ is exhibited in Fig. 4c. Three peaks can be obtained after deconvolution. Among those 226.1 eV corresponds to S 2s of MoS_2_. The two main peaks of Mo 3d_5/2_ (229.3 eV) and Mo 3d_3/2_ (232.4 eV) are characteristics of MoS_2_. Sulfur species of pristine MoS_2_ are shown in Fig. 4d. The binding energies of 162.0 and 163.2 eV correspond to the S 2p_3/2_ and S 2p_1/2_ of MoS_2_, respectively^37^. As for MG3 composite structure, the above binding energies are all slightly larger than that of pristine MoS_2_. This result indicates that there exists a certain interaction between MoS_2_ and rGO sheets in the composited structure, driven by a probable electrons transfer between firmly contacted MoS_2_ and rGO sheets. In the composite, the co-electron cloud could form between S atom and its adjacent carbon layer. The interaction of electrons between MoS_2_ and rGO could dramatically enhance the conductivity of the composite and is conducive to the corresponding HER process. Generally, there exists the strong Coulomb interaction between the inner electrons and nucleus in the atom, while the outer electrons can also apply the shielding effect to the inner electrons. Therefore, the decrease of electron concentration of outer electrons will lead to the reduction of the corresponding shielding effect and thus increase the binding energy of the inner electrons for the existence of penetration effect, resulting in the corresponding XPS peaks shift to a higher energy direction. Obviously, the above XPS surveys reveal that electrons can transfer form MoS_2_ to rGO, which is also consistent with the calculated results that the directional transfer of electrons from MoS_2_ to graphene network and a built-in electric field establishment directed from MoS_2_ to graphene. A further quantitative elements analyses by XPS reveal that the oxygen percentages decreased from 5.31, 4.83, 4.11 to 3.27% and the atomic ratio of S:Mo also reduced from 2.27, 2.23, 2.12 to 2.07 as the synthesis temperatures increasing for MG1 to 4. These changes mean that the synthesis temperatures have a direct link to the incorporated oxygen and the unsaturated sulfur atoms, which will inevitably influence the electronic structure of MoS_2_ and exert a direct influence on the HER activity. This will be discussed in details in the following sections.

**Figure 5:**
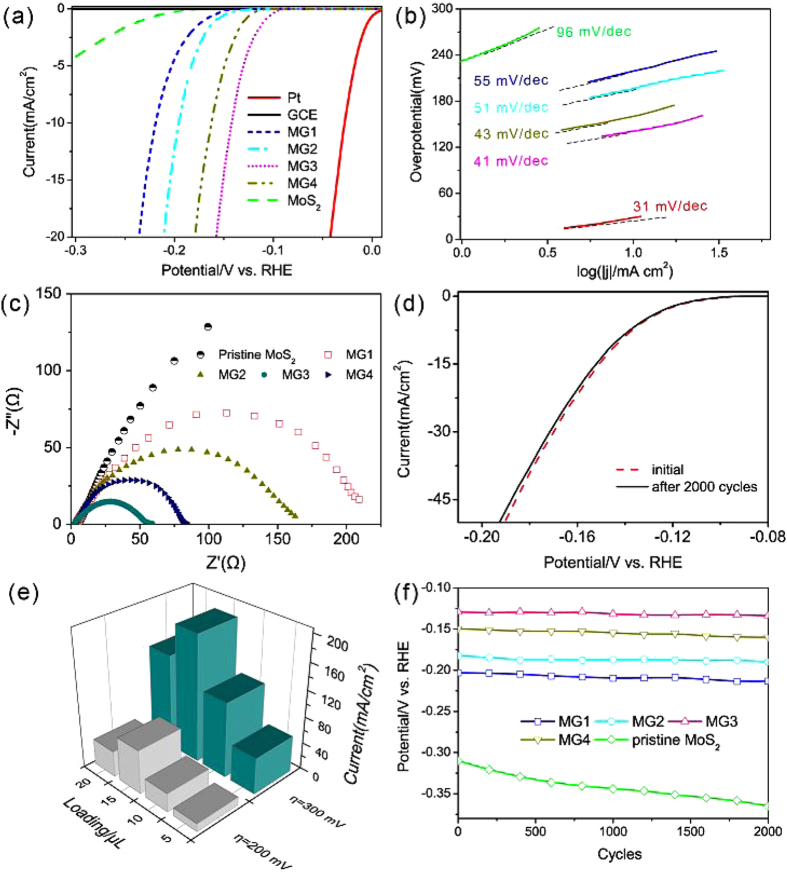
(**a**) Polarization curves obtained with various catalysts. (**b**) The corresponding Tafel plots. The linear regions of the Tafel plots are fitted into the Tafel equation (η = blog(j) + a, where b is the Tafel slope). (**c**) Nyquist plots of EIS for various electrocatalysts at the modified GCEs. (**d**) Stability test of the MG3 electrocatalyst. Negligible HER current was lost after 2000 cycles of CV. (**e**) Current densities for MG3 composite with different loadings at overpotentials of 200 and 300 mV. (**f**) Potential values for 2000 CV sweeps for pristine MoS_2_ and MG composite structures.

Figure 5a,b show polarization curves and the corresponding Tafel plots, respectively. It is shown that Pt exhibits a near zero overpotential and has the highest HER activity compared with the other catalysts owing to a high H_ads_ coverage. The HER process on the Pt surface generally follows the Volmer-Tafel mechanism as described below. The rate-limiting step is corresponding to recombination step and it has the Tafel slope of 31 mV/decade. The polarization curve of GCE is also plotted, while the trivial current indicates that the GCE is catalytically inactive for the HER. It can be seen that much lower onset overpotentials (η) are obtained for all MG catalysts compared to pristine MoS_2_. Among composite structures, MG3 presents the lowest onset η of about 110 mV, implying a more superior HER activity and the cathodic current density rises rapidly under a more negative potential. The optimal catalytic performance of MG3 composite is attributed to the synergetic effect of the optimal intrinsic conductivity and the excellent conductive network provided by rGO network. The corresponding Tafel plot indicates that the suitably crystallized MG3 possesses the smallest Tafel slope than the poorly crystallized MG1 and MG2 or highly crystallized MG4. This proves that the crystallinity of MoS_2_ should be controlled in a reasonable level for a higher HER performance, since a poor crystallinity leads to the decreased conductivity for the suppressed electron transfer along the basal surfaces, which ends in a weakened HER activity. However, even if the improved crystallization can enhance the conductivity with increase of temperature, the inherent incorporated oxygen and sulphur atoms will gradually decrease as analyzed by XPS. In this case, the decrease of the two atoms that play promotion roles in the corresponding HER routine will lead to a subdued intrinsic conductivity in reverse which will be discussed later. Hence, a moderate crystallinity degree with quasi-crystallinity structure is more beneficial for HER.

The kinetic of the HER depends upon the electrochemical conditions and the corresponding Tafel slope is often utilized to elucidate the dominant mechanisms involved in the HER routine. There are two possible reaction steps in acidic aqueous. First, the discharge reaction^38^,


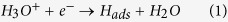


second, the combination reaction:


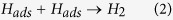


or the desorption reaction:


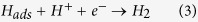


Generally, a fast discharge reaction (1) followed by a rate-limiting combination reaction (2) leads to a Tafel slope of ∼30 mV dec^−1^. When (1) is fast and followed by a slow electrochemical desorption reaction (3), a Tafel slope of ∼40 mV dec^−1^ is obtained. If (1) is rate-limiting or the surface coverage is close to one, the Tafel slope is ∼120 mV dec^−1^. The Tafel slope values obtained from Fig. 5b are ∼96, ∼55, ∼51, ∼41, and ∼43 mV dec^−1^ for pristine and MG1 to 4 composites, respectively. It is noteworthy that the Tafel slope of ∼41 mV dec^−1^ for MG3 composite suggests that desorption is the rate-limiting step.

EIS can be further utilized to study the interface reactions and electrode kinetics of pristine MoS_2_ and MG composites in HER processes. The Nyquist plots of different structures are given in Fig. 5c. The MG3 electrode shows the smallest radius of semi-circle in the Nyquist plots, implying the lowest contact and charge-transfer resistance (R_ct_) for the MG3 electrode. The obtained semicircles are also in accord with the HER activity shown in Fig. 5a. It is known that a lower R_ct_ corresponds to a faster reaction rate. In this case, a faster charge transfer during HER reaction with a moderate crystallinity and rGO incorporation contribute to a superior HER activity of the MG3 composite for the achievement of both structural and electronic synergistic effect for the prominent HER performance. We also cycled the catalysts continually for 2000 cycles to probe HER stability. In this process, the catalyst poisoning or delaminating from the electrode may cause a certain activity loss of catalytic. As shown in Fig. 5d, at the end of cycling, the current density of MG3 composite catalyst decreases slightly in comparison with initial value after the continual cycling, implying that the synthesized MG3 composite exhibits good long term stability of HER activity. The dependence of different MG3 loadings on GCE and the HER activity is shown in Fig. 5e. It is shown that the optimal loading of MG3 is 15 mL with the current densities of 66 and 191 mA cm^−2^ at 200 and 300 mV, respectively. Besides, the potential values of various structures recorded at the same current density of 5 mA cm^−2^ for 2000 sweeps are displayed in Fig. 5f. It can be seen that all MG composites show good durability with negligible increases of overpotential in comparison with pristine MoS_2_. We consider that the hybrid structures of MG can enhance the durability, which can efficiently hinder the degradation of catalysts in HER routines.

## Discussion

**Figure 6:**
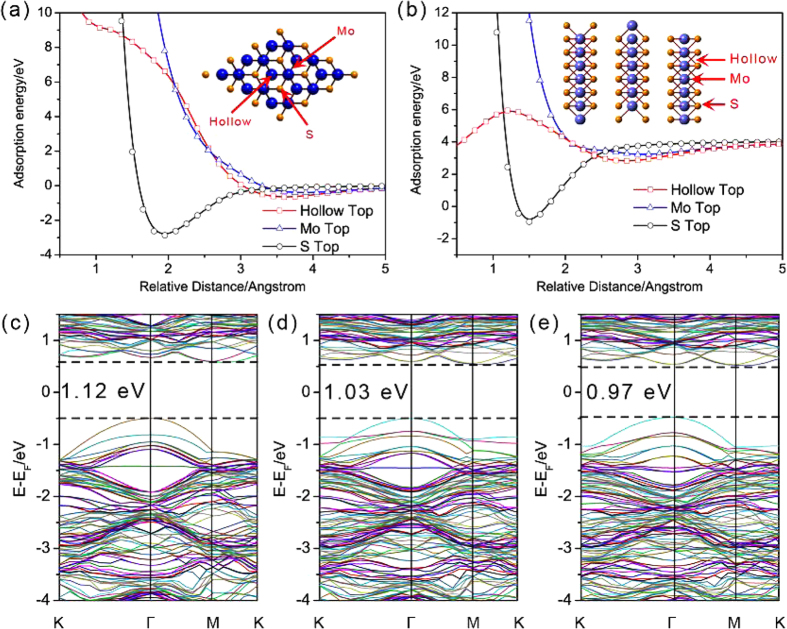
(**a**,**b**) are the energy profiles as a function of relative distance (Å) of S and O atom above the three sites for basal MoS_2_, respectively. The three sites are hollow top site, Mo top site, and S top site. (**c–e**) are the band structures of pristine and S/O atoms absorbed MoS_2_, respectively.

From the above XPS and EIS analyses, it is shown that the incorporation of oxygen and sulfur atoms with moderate crystallinity can realize the modulation of conductivity for a better HER performance. Next, we will analyze how it influences the electronic structure of the specific structures when in S/O rich conditions. For the purpose of excluding the effect of the different adsorption sites between the pristine and S/O incorporated structures, the following adsorption analyses are conducted based on the most stable adsorption configurations for the two structures. The related adsorption sites considered include the S/Mo top and hollow top sites as shown in the insert Fig. 6a,b. The calculated adsorption energies as a function of relative distance clearly show that the most stable sites of S and O atoms prefer to bond are S top sites with distances of 1.95 and 1.51 Å, respectively. The calculated results reveal that the absorption energies of the two sites for the corresponding structures are all less than zero, implying energetically favorable adsorption properties of S and O. These results are consistent with the above XPS analyses. As shown in Fig. 6d,e, the S/O incorporated MoS_2_ exhibits a narrower bandgap (1.03/ 0.97 eV) in comparison with pristine structure (1.12 eV), implying an easier electron transfer from valance band maximum (VBM) to conduction band minimum (CBM) and thus the superfluous of S/O can lead to an easier electrons transfer. Also, a heavier incorporation can further reduce bandgap and thus regulates band structure. The reduced bandgap ascribes to the incorporation of S/O atoms can enhance hybridization effect between Mo d-orbital and S/O p-orbital. This means that the impurities levels from the absorbed S/O can effectively reduce band gaps. According to the present work, the effective directional transfer of electrons can form a built-in electric field directed from MoS_2_ to graphene. This electrical coupling will facilitate the following rapid electrons transfer from electrode to rGO and then to MoS_2_, forming an excessive negative charge density which will effectively prompt ΔG shift to the thermo-neutral state and finally facilitate the overall HER routine.

**Figure 7:**
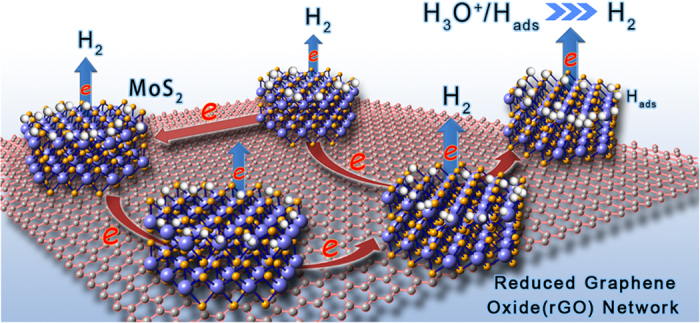
Schematic diagram of the synergistic effect for MG composites in HER routine. The red network around carbon atoms represents the transfer of electrons, which acts as a conductive electron “highway” and provides an excessive negative charge environment.

According to the previous reports^39^, the disordered structure can provide more unsaturated sulphur atoms as active sites for HER, but the collapse of 2D electron conjugation would restrain the electron transfer along the basal surfaces with poor crystallinity, which results in a low hierarchy conductivity and a weak overall HER activity. In this work, the improved electrical conductivity and the formation of the excessive negative charge would play an important role in achieving a higher HER efficiency for the MG composites compared with pristine MoS_2_. We attribute the optimized performance of MG composites to the strong chemical and electronic synergetic effects between rGO network and MoS_2_. On one hand, the confined growth of highly dispersed MoS_2_ on rGO is free of aggregation^40^. The small size and highly dispersed MoS_2_ on rGO afford a large amount of accessible edges that can be used as active sites for HER. For another, electrical coupling to the graphene also make a rapid electron transfer from electrodes to rGO and then to MoS_2_, forming an excessive negative charge density to improve the Gibbs free energy and facilitate the HER routine (see schematic of Fig. 7). Therefore, the 2D rGO network is believed to play a crucial role as a charge transfer “highway” for an evolved electrocatalytic activity. As expected, the obtained MG composites show excellent catalytic activity of HER characterized by higher current densities and lower onset potentials than that of the pristine MoS_2_ structure.

On the premise of the composited with rGO, the controllable crystallinity of MoS_2_ and the corresponding different degrees of O atom incorporation as well as Mo/S ratios provide an opportunity to further regulate the structure and electron configuration. As the degeneration of crystallinity, more O/S atoms are generated and act as active sites for HER routine and the incorporation of the two kinds of atoms can effectively reduce the bandgap of MoS_2_, leading to an improved conductivity. But the damage of 2D electron conjugation of the disordered MoS_2_ is a disadvantage factor for the better conductivity and limit the shift of ΔG to thermo-neutral state, which is the crux for the improvement of HER performance. Therefore, the optimized conductivity and the incorporated O/S should be reasonably regulated by modulating the degree of crystallinity for the composite MoS_2_, which will eventually lead to a better HER performance. In brief, we conducted the first-principles calculations and the mechanisms of elections’ transfer were studied detailedly to analyze and interpret the corresponding properties from a theoretically perspective. Credible conclusions achieved and the corresponding analytical methods acted as a bridge between experimental results and calculation conclusions. We believe this work will provide valuable theoretical guidances for the research of hybrid structures in the future.

## Methods

### Synthesis of MoS_2_/rGO composite

The MoS_2_/rGO composites were synthesized by a one-step hydrothermal reaction of Na_2_MoO_4_·2H_2_O and NH_2_CSNH_2_ in aqueous solution containing GO. For a typical synthesis of the MoS_2_/rGO, 100 mg GO powders were dispersed into 60 mL distilled water and ultrasonicated until fully dispersed. Afterwards, 1 g of Na_2_MoO_4_·2H_2_O and 1.2 g of NH_2_CSNH_2_ were dissolved into the above suspensions meanwhile and kept stirring. Then, the mixture solution was transferred into a Teflon-lined autoclave and heated at 140, 160, 180 and 200 °C. The obtained samples were labelled as MG1 to 4, where M and G refer to MoS_2_ and rGO, respectively. After 24 h reaction, the black products were scraped and washed carefully with distilled water and anhydrous ethanol in sequence, and then dried in an oven at 60 °C for 12 h. Pristine MoS_2_ was prepared using the same processes as MG3 while no GO was added in.

### Characterization of MoS_2_/rGO nanostructures

In this work, X-ray diffraction (D8 Advance/ BRUKER AXS GMBH) with Cu-Kα radiation (λ = 0.1541 nm) was used to characterize the different samples. The diffraction data were collected in the 2θ scanning ranging from 10° to 70° with 0.02° per step. The morphologies of the samples were obtained by field emission scanning electron microscopy (FESEM, JEOL-JSM-6700F) at an accelerating voltage of 20 kV and transmission electron microscopy (TEM, JEOL-JEM-2100) at an accelerating voltage of 200 kV. The sample used for TEM characterization was prepared by dropping the colloidal solution to a holey carbon-coated copper grid, which was then dried in air. Raman spectra analyses were carried out by a Jobin-Yvon LabRAM HR 800 micro-Raman spectrometer. X-ray photoelectron spectrometry (XPS) analyses were performed by ESCALAB 250Xi.

### Electrochemical studies

Electrochemical measurements were performed on a glassy carbon electrode (GCE) in 0.5 M H_2_SO_4_ solution. 2 mg MG composite was dispersed in a 1 mL 4 : 1 v/v water–ethanol, and then blended with 20 μL 5 wt% Nafion. After 30 min sonicated, a homogeneous ink formed and the mixture was casted onto the GCE for the following electrochemical measurements. 5 μL of the dispersion was loaded onto a glassy carbon electrode. Different loadings of the catalyst were obtained by repeatedly adding mixture until it reached the required values. Linear sweep voltammetry (LSV) method with scan rate of 5 mV s^−1^ was used to evaluate the HER performance of different catalysts. Catalytic modified GCE acted as the working electrode. Pt wire served as the counter electrode and saturated calomel electrode (SCE) acted as the reference electrode. Prior to all electrochemical measurements, the electrolyte solutions were purged with N_2_ for 1 h to remove the oxygen completely.

### Computational details

In this section, first-principles calculations were performed based on the DFT together with the projector-augmented wave (PAW) potential implemented in the Vienna ab initio simulation package (VASP). The valence electrons considered were: Mo (p^4^s^5^d^4^), S (s^2^p^4^), O (s^2^p^4^) and C (s^2^p^2^), respectively. The generalized gradient approximation (GGA) functional of Perdew, Burke and Ernzerhof (PBE) was used to deal with the exchange and correlation potentials. Basal calculations were performed with a 3 × 3 × 1 supercell. The spacing of 15 Å between two dimensional single layers of pristine MoS_2_ and MoS_2_/graphene was used to avoid interlayer interactions. A 5 × 5 × 1 Monkhorst-Pack k-point sampling for the Brillouin zone k-point mesh and a 500 eV cutoff energy were used for the calculations.

## Additional Information

**How to cite this article**: Li, H. *et al.* Charge-Transfer Induced High Efficient Hydrogen Evolution of MoS_2_/graphene Cocatalyst. *Sci. Rep.* **5**, 18730; doi: 10.1038/srep18730 (2015).
